# Supplementary material for: Patterns of Intron Gain and Loss in Fungi
Source: PLoS Biol. 2004 Nov 30;2(12):e422. doi: 10.1371/journal.pbio.0020422 (PMC532390; doi:10.1371/journal.pbio.0020422)
Supplement: Table S1 — Also available at http://genes.mit.edu/NielsenEtAl/. (4.3 MB ZIP). [file pbio.0020422.st001.zip › NielsenEtAl/html/1444.html]

AN1209.1.NCU02967.1.MG05617.1.FG01338.1


```
 CLUSTAL W (1.82) Multiple Sequence Alignments - Introns Inserted


Sequence 1: AN1209.1	237 aa
Sequence 2: NCU02967.1	237 aa
Sequence 3: MG05617.1	242 aa
Sequence 4: FG01338.1	226 aa
Alignment Length: 248 aa
Number Identitical Residues: 89 aa
Alignment Score (without introns) 5039


MG05617.1 	-MDTENKFAIHAACREGRY1AAVESLLN0VDPKLANRK~DDDGRLPIHWAVSSNQFEITN
NCU02967.1	--MAEDKFAIHAAARDGKA1SIVESLLN0ANPKLAQRK~DDDGRLPIHWACSYNRKEVVE
FG01338.1 	-MEKEDKFPLHTAAREGRV1PVAEGLL-~--------K0DDDGRYPIHWAASSNNLEIVQ
AN1209.1  	MATTDSKFPLHEAAREGKL1SLAESLLN0ANPKLATTK~DDDDRLPIHWAVAYNHLPIVE
          	    :.**.:* *.*:*:  . .*.**.  ... :  * ***.* ***** : *.  :.:

MG05617.1 	LLVAQKNFDPDVQ~DDSGWTPLMIAVSIKDG--EKLVDLLLGKGADVNEKT1NMDL~LYT
NCU02967.1	LLVNQKGFDPDVE0DDMGWTPFMISASVKDS--DAIIDLLLSRGADINQTN~HQSQ~TAL
FG01338.1 	LLANQRSFDADVQ0DGSGWSPLMISASVPES--EAVLKLIISKDADVNLKN1FTGQ0TAL
AN1209.1  	LLVSTKDFDPDVE0DGSCWTPLMIAASLRNAEGDPIIELLLRKGADVNAKS~ITGQ0NAL
          	**.  :.**.**: *.  *:*:**:.*: :...: ::.*:: :.**:* ..   .     

MG05617.1 	EDNSGQ0NNLDVARKLLDQK---PPASARVRDRRGQYALHRAAAVGSTPMVNLLIGQGKS
NCU02967.1	HFIASK~NNIDLARKLLSPDMKPKPASVRVKDKRGQYPLHRAAAIGSVPMINLLLQH-KS
FG01338.1 	HFVASK~KNLDIARILIDSG-----ASTRVRDRRGQYPIHRAAAVGSVPMVNILLKN-RS
AN1209.1  	HFATSK~ANITTVRTLLEN-----KCSARVKDKRGQLALHRAAAIGSTPIIQLLLKEGRS
          	.  :.:  *:  .* *:.       .*.**:*:*** .:*****:**.*::::*: ..:*

MG05617.1 	PLNATDSDGQTALHHAIAEGHG1DTAVALLKAGAETDKKDADGNLAIDLAPDRE~VRKYI
NCU02967.1	PINASDNAGYTPLHHAVAEGHG~HAAVALLKAGAETDKKDMDGYLALDLAPDKD~VRRFI
FG01338.1 	PLNPTDNEGYTPLHHAIAEGHG1DTAVALLKEGADFTLKNSAEELAMDLAPDKE0VRRYI
AN1209.1  	PVNATDNDGLTALHHAISEGHG~DAALTLLKAGAEADKKDASGTLAIDMAPDKK0VRNYI
          	*:*.:*. * *.****::**** .:*::*** **:   *:    **:*:***:. **.:*

MG05617.1 	EQVAEREGVDLPSGSSA
NCU02967.1	EKEAEKEGIEL------
FG01338.1 	LQGAEREGIEMSG----
AN1209.1  	LQAAEREGIEL------
          	 : **:**:::
```
